# Antiviral roles of eosinophils in asthma and respiratory viral infection

**DOI:** 10.3389/falgy.2025.1548338

**Published:** 2025-02-28

**Authors:** Hisashi Sasaki, Jun Miyata, Akihiko Kawana, Koichi Fukunaga

**Affiliations:** ^1^ Division of Infectious Diseases and Respiratory Medicine, Department of Internal Medicine, National Defense Medical College, Saitama, Japan; ^2^ Division of Pulmonary Medicine, Department of Medicine, Keio University School of Medicine, Tokyo, Japan

**Keywords:** asthma, asthma exacerbation, eosinophil, interferon, antiviral activities

## Abstract

Eosinophils are immune cells that are crucial for the pathogenesis of allergic diseases, such as asthma. These cells play multifunctional roles in various situations, including infection. They are activated during viral infections and exert antiviral activity. Pattern recognition receptors, toll-like receptor 7 and retinoic acid inducible gene-I, are important for the recognition and capture of RNA viruses. In addition, intracellular granule proteins (eosinophil cationic protein and eosinophil-derived neurotoxin) and intracellular nitric oxide production inactivate and/or degrade RNA viruses. Interestingly, eosinophil-synthesizing specialized pro-resolving mediators possess antiviral properties that inhibit viral replication. Thus, eosinophils may play a protective role during respiratory virus infections. Notably, antiviral activities are impaired in patients with asthma, and eosinophil activities are perturbed in proportion with the severity of asthma. The exact roles of eosinophils in RNA virus (rhinovirus, respiratory syncytial virus, and influenza virus)-induced type 2 inflammation-based asthma exacerbation remain unclear. Our research demonstrates that interferons (IFN-α and IFN-γ) stimulate human eosinophils to upregulate antiviral molecules, including guanylate-binding proteins and tripartite motifs. Furthermore, IFN-γ specifically increases the expression of IL5RA, ICAM-1, and FCGR1A, potentially enhancing cellular responsiveness to IL-5, ICAM-1-mediated adhesion to rhinoviruses, and IgG-induced inflammatory responses, respectively. In this review, we have summarized the relationship between viral infections and asthma and the mechanisms underlying the development of antiviral functions of human and mouse eosinophils *in vivo* and *in vitro*.

## 1 Introduction

Eosinophils are a type of granulocytes that contain abundant intracellular granule proteins that can be stained using acidic aniline dyes (1). This cell type performs various cellular functions, including degranulation, reactive oxygen species (ROS) production, cytokine release, inflammatory lipid mediator synthesis, and EETs (Eosinophil extra-cellular traps). Eosinophils play a central role in the pathogenesis of predominantly allergic disorders, as well as collagen vascular diseases, neoplastic conditions, and infectious diseases, acting as effector cells that mediate inflammatory responses in local tissues (2–4). In contrast to pro-inflammatory eosinophils that promote allergic inflammation, a subset known as tissue-resident eosinophils has been identified, which contributes to maintaining physiological homeostasis (5). This cell population is involved in glucose metabolism; prevention of obesity; muscle regeneration; immunoglobulin A production; induction of regulatory T cell differentiation; and synthesis of specialized pro-resolving mediators to suppress inflammatory responses and promote the resolution of inflammation (6–9). Tissue-resident eosinophils with these homeostatic cellular functions reside mainly in the gastrointestinal tracts and substantially in respiratory and lower urinary tracts (8, 9). Interestingly, eosinophils also participate in the clearance of microorganisms *in vivo* and *in vitro*. They respond to parasite invasion by degranulating (2, 10). In addition, they exhibit phagocytic activity via their granule proteins against microorganisms, including Staphylococcus aureus and Escherichia coli (11–13). Recent studies have demonstrated that eosinophil ETosis (EETosis) in which an eosinophil lyses and releases their DNA components and intracellular proteins is involved in bacteriostatic activity against S. aureus and *Aspergillus fumigatus* (14, 15). Additionally, previous reports have demonstrated the antiviral role of eosinophils in humans and mice. Eosinophil cationic protein (ECP) and eosinophil-derived neurotoxin (EDN), major granule proteins of eosinophils, function as ribonucleases that degrade RNA viruses (16). Human eosinophils can capture influenza virus and respiratory syncytial virus (RSV) and inactivate them (17). Interestingly, eosinophils isolated from asthma patients have lower antiviral abilities than those isolated from healthy subjects (17). In addition, asthma severity correlates with reduced capacity of capturing viruses. Experimental viral infection models using mice have shown eosinophil-mediated protection against influenza virus, parainfluenza virus, and RSV (18–20). In contrast, blood eosinophils from patients with severe asthma have been reported to suppress IFN-α production from airway epithelial cells and the functions of plasmacytoid dendritic cells (pDC) cells, contributing to asthma exacerbation in viral infections (21). Another study demonstrated that coculture of eosinophils with BEAS-2B cells induced transforming growth factor β secretion, which may suppress human rhinovirus (HRV)-induced IFN expression (22). These findings highlight the biological significance of the pro-viral or anti-viral roles of eosinophils.

In this review, we have focused on the antiviral activity of eosinophils. We have discussed the immunological mechanisms underlying their antiviral activities that are mediated by specific molecules and their receptors. The relevance and involvement of eosinophils in viral infections in asthma have also been discussed.

## 2 Pathophysiological roles of respiratory viral infection in asthma

### 2.1 Characteristics of eosinophilic inflammation in asthma

Asthma is a chronic airway inflammation that causes paroxysmal cough, dyspnea, wheezing, and chest discomfort (23). Among patients with asthma, 50-70% patients present with type 2 inflammation characterized by eosinophil accumulation in the airway (24). Type 2 cytokines include interleukin-4 (IL-4), IL-5, and IL-13, which are optimal therapeutic targets to treat severe eosinophilic asthma (25). From the perspective of viral infections, elevated fractional exhaled nitric oxide levels and sputum eosinophil counts are associated with an increased risk of future virus-induced exacerbations (26). T helper type 2 (Th2) cells and group 2 innate lymphoid cells (ILC2) mainly produce type 2 cytokines (27). IL-5 prolongs eosinophil survival and activates eosinophil functions, including degranulation, superoxide generation, and cytokine release, leading to airway epithelium injury with increased airway hyper-responsiveness (AHR) (28). Also, EETosis causes mucus plug formation and deposition of Charcot-Leyden crystals (3, 29–31). The numbers of EET^+^ eosinophils and ILC2s were significantly elevated in severe asthma, with a positive correlation between these cell populations. Furthermore, mice injected with EETs exhibited a significant increase in eosinophil and ILC2 counts (32). Eosinophils isolated from patients with asthma have altered expression patterns of surface antigens, including CD69, indicative of their activated status (33). CD69 is a marker of tissue-resident T cells involved in the production of type 2 cytokines (34), suggesting that CD69-high eosinophils may contribute to the maintenance of tissue autonomy. Approximately 5%–10% patients with asthma have severe disease, with resistance to standard treatments and are on other treatments such as inhalation of corticosteroids and add-on agents. Blood eosinophils display EETosis more frequently in patients with severe asthma than in those with mild to moderate asthma (35). Airway eosinophils obtained from patients with atopic asthma also induce EETosis (36). In addition, eosinophils isolated from patients with severe asthma or eosinophilic chronic rhinosinusitis, its comorbidity, showed dysregulated fatty acid metabolism (37, 38). These findings are indicative of cellular changes in eosinophils due to systemic and/or local inflammatory milieu in severe asthma. Recently, biologics targeting type 2 cytokines have become available for the treatment of severe asthma. IL-5, a strong activating cytokine for eosinophils, is an optimal target for reducing eosinophilic inflammation. Mepolizumab, an IL-5-neutralizing antibody, and benralizumab, an IL-5 receptor α (IL-5Rα)-targeting antibody, dramatically reduced the number of eosinophils in the blood and airways (39, 40). Anti-IL-5 treatment restores rhinovirus-induced IFN-α secretion by pDCs in patients with asthma (21). Omalizumab, an IgE-neutralizing antibody, also enhances IFN-α responses and reduces Fc*ε*RIα protein expression in pDC, providing evidence that these effects are related (41). These biologics have been shown to reduce asthma exacerbation, frequently caused by viral infection, and spare the intake of oral corticosteroids with long-term safety (42–49). These therapeutic effects indicate that eosinophils play an important role in asthma as an inflammatory immune cells.

### 2.2 Roles of viral infection in asthma

Viral infections account for 40%–80% cases of asthma exacerbations (50, 51). Among these, rhinovirus, RSV, and influenza virus account for most cases of asthma exacerbations. Parainfluenza viruses, human metapneumoviruses, and enteroviruses also induce asthma exacerbation at low frequencies (50, 51). Rhinoviruses and respiratory syncytial virus (RSV) suppress IFN production by airway epithelial cells and basal cells, contributing to asthma exacerbations (52, 53). In murine models of asthma, infections with these viruses and influenza viruses also induce eosinophilic airway inflammation. Elevated levels of type 2 cytokines, eosinophil-derived granule proteins, and leukotrienes have been detected in the airways of asthmatic patients infected with these viruses (54–58). In the following sections, we will discuss previous reports on the pathogenic relationship between viral infections and asthma.

### 2.3 Rhinovirus

Rhinoviruses most frequently cause respiratory tract infections and subsequent asthma exacerbations. Rhinovirus is correlated with asthma exacerbations in school-aged children in age-stratified time-series analysis (59). The ability to induce apoptosis for inhibiting viral replication is reduced in airway epithelial cells of asthma patients infected with rhinoviruses (52) in general, rhinovirus infection causes the production of type 1 interferons (IFN), IFN-α and IFN-β, and type 3 interferon, IFN-*λ*, in airway epithelial cells. However, the levels of IFNs in airway epithelial cells of patients with asthma were decreased compared with healthy subjects and those levels correlate negatively with blood eosinophil count and serum IL-4 concentration (52, 54). In contrast, human airway epithelial cells infected with rhinoviruses secrete Regulated on Activation Normal T Cell Expressed and Secreted (RANTES), resulting in accumulation of eosinophils in the airway (60). Rhinovirus infection induces pulmonary type 2 inflammation in mice. Compared to that observed after rhinovirus type A infections, rhinovirus type C infection increases the production of IL-5, IL-13, IL-25, IL-33, and thymic stromal lymphopoietin (TSLP), with abundant eosinophil infiltration in the airways (48). Airway eosinophilic inflammation did not occur in Roraflox/flox Il7r-Cre mice lacking ILC2, indicative of the importance of ILC2 in this model (61). Compared to that observed in non-sensitized mice, infection with rhinovirus type 1B resulted in eosinophilic inflammation with increased expression of IL-33 and IL-13 in a house dust-sensitized asthma model (62). These results suggested that rhinovirus infection induces type 2-high airway inflammation, which is mainly dependent on ILC2 in mice. In bronchoalveolar lavage fluid (BALF) from 11 rhinovirus-infected patients with moderate asthma, the concentrations of IL-33 and IL-25 correlated positively with those of IL-4, IL-5, and IL-13, suggesting the involvement of innate immunity-related type 2 inflammation (63, 64). ILC2 levels in BALF are predominantly higher in rhinovirus-infected asthma patients than in healthy subjects (65). In addition, the ILC2:ILC1 ratio increased in patients with asthma and correlated positively with the clinical score of asthma exacerbations and the concentration of type 2 cytokines in nasal mucosal lining fluid (65). In total, these findings indicated that rhinovirus infection in asthma diminishes type I IFN production and involves ILC2-mediated type 2 inflammation in human.

### 2.4 RSV

RSV infection, the major cause of bronchiolitis in children, is closely associated with the development of childhood-onset asthma. Among children who were infected with RSV within the first year of their lives, 30% of the children were diagnosed with asthma and/or wheezing within 7 years after infection (66). Non-structural protein 1 (NS1) of RSV inhibited IFN production with impaired IFN-related signaling and reduced antiviral immunity in A549 cells, a human epithelial cell line (53). In murine experiments, NS1 increased serum levels of TSLP and OX40l, inhibited the induction of regulatory T cells, and disrupted immune tolerance mechanisms (55). In an ovalbumin (OVA)-induced asthma model, RSV infection enhanced AHR, a persistent mucus production, and subepithelial fibrosis (67). In the RSV-infected asthma model, high levels of eosinophilic extracellular DNA were detected in the BALF, which is indicative of EETosis induction in the airway (68). Overall, these results suggested that RS virus infection can exacerbate asthma in mice. When stimulated with major basic protein (MBP), eosinophil peroxidase (EPO), and eosinophil granule proteins, RSV-infected human type II alveolar epithelial cells showed necrotic changes due to their cytotoxicity (57). The concentrations of ECP and EDN, other types of eosinophil granule proteins, and eosinophil chemoattractants (MIP-1α and RANTES) in nasal secretions of children with RSV-induced bronchiolitis were higher than those in nasal secretions of normal subjects (69, 70). Also, high levels of cysteinyl leukotrienes (cys-LTs) were detected in the sputum of asthma patients with RSV infection (56). Thus, eosinophils in patients with RSV infection can enhance airway inflammation and contribute tissue damage in human.

### 2.5 Influenza virus

Influenza viruses are RNA viruses belonging to the Orthomyxoviridae family that cause seasonal infections in humans (71). A previous report has shown that influenza A virus was the most common cause for asthma exacerbation in 79 hospitalized patients with asthma, suggesting a close association of this virus with asthma (72). Animal experiments using asthma models have demonstrated an association between influenza virus and asthma. The OVA-sensitized asthma model of mice with influenza virus infection showed higher levels of OVA-specific IgE and AHR with airway epithelial injury than uninfected mice (73). House dust-sensitized mice infected with influenza virus showed increased AHR due to airway epithelial cell-derived IL-33-mediated responses (74). House dust-sensitized mice infected with influenza virus (X31 H3N2) showed increase in the number of ILC2 in the airway, along with high levels of IL-5 and IL-13 in BALF, especially upon virus clearance (58). Interestingly, infection with influenza type A virus induced the production of large amounts of IFN-γ in the lungs during the early phase of infection and enhanced type 2 inflammatory responses in the late phase (75). These findings emphasize the importance of influenza virus infection as a cause of asthma exacerbation with type 2 inflammation in mice. In human, among 34 children aged 2–11 years infected with H1N1 influenza A virus, 21 patients with pneumonia had higher serum levels of IFN-γ and IL-5 than patients without pneumonia. Also, patients with severe pneumonia had significantly higher serum levels of IL-4, IL-5, and IL-13 than those with mild pneumonia (76), indicative of the role of influenza virus infection in airway type 2 inflammation. In contrast, lower rates of pneumonia, mechanical ventilation, and mortality were observed in asthma patients infected with H1N1 influenza virus than in non-asthmatics (77, 78), suggesting that type 2 inflammation in the airways might provide protection from fatal H1N1 infection in human.

### 2.6 Parainfluenza virus

Parainfluenza virus is a single-stranded RNA virus belonging to the paramyxovirus family. The concentrations of cysLTs in the sputum of patients infected with parainfluenza during mild asthma exacerbations were higher than those in the sputum of patients with non-viral asthma exacerbations (79). ILC2 remains in the lungs of mice infected with parainfluenza virus after the virus has cleared and may be associated with the development of asthma (80). Studies evaluating the relationship between viral infection, asthma, and type 2 inflammation are summarized in Table 1.

**Table 1:** Relationship between viral infection, asthma and type 2 inflammation.

Virus	Animal	Asthma type	Tissues	Type 1 inflammation	Type 2 inflammation	Reference
Rhinovirus	Human	Moderate asthma	BEC	IFN-β↓	ND	(52)
	Human	Atopic asthma childlen	BEC	IFN-β↓, IFN-*λ*↓	IL-4↑, IgE↑	(54)
	Human	Moderate asthma	BALF	ND	IL-4↑, IL-5↑, IL-13↑, IL-25↑, IL-33↑	(63)
	Human	Mild or moderate asthma	BALF	ND	IL-4↑, IL-5↑, IL-13↑, IL-33↑	(64)
	Human	Moderate asthma	BALF	ND	ILC2↑	(65)
	Mouse	House dust sensitized model	BALF	ND	IL-5↑, IL-13↑, IL-25↑, IL-33↑, TSLP↑	(61)
	Mouse	House dust sensitized model	BALF, Lung	ND	IL-13↑, IL-33↑	(62)
RSV	Mouse	OVA sensitized model	Serum, BALF	IFN-γ↓, IL-10↓	IL-4↑, IL-5↑, TSLP↑	(55)
	Mouse	OVA sensitized model	BALF	IFN-γ↑, TNF-α↑	IL-5↑	(67)
	Mouse	OVA sensitized model	BALF	IFN-γ↓	ND	(68)
Influenza virus	Mouse	OVA sensitized model	BALF	ND	IgE↑	(73)
	Mouse	House dust sensitized model	BCC, Type II alveolar cells	IFN-β↓	IL-33↑	(74)
	Mouse	House dust sensitized model	BALF	ND	IL-5↑, IL-13↑	(58)
Parainfluenza virus	Human	Mild asthma	BALF	ND	Cys-LTs	(79)
	Mouse	Parainfluenza virus infection model	Lung	ND	ILC2↑	(80)

BALF, bronchoalveolar lavage fluid; BCC, bronchial ciliated cells; BEC, bronchial epithelial cells; Cys-LTs, cysteinyl leukotrienes; IFN, interferon; IgE, immunogloblin E; IL-4, interleukin-4; IL-5, interleukin-5; IL-10, interleukin-10; IL-13, interleukin-13; IL-25, interleukin-25; IL-33, interleukin-33; ILC2, group 2 innate lymphoid cells; OVA, ovalbumin; RSV, respiratory syncytial virus; TNF-α, tumor necrosis factor-α; TSLP, thymic stromal lymphopoietin; ND, no data.

## 3 Eosinophils in patients with COVID-19 and asthma

### 3.1 Relationship between COVID-19 and asthma

Coronavirus disease 2019 (COVID-19) is an emerging infection caused by severe acute respiratory syndrome coronavirus 2 (SARS-CoV-2) that has become a global threat since its outbreak in Wuhan in December 2019. Coronaviruses, including SARS-CoV-2, are RNA viruses that cause mild to severe respiratory failure. Although some studies have reported their involvement in asthma exacerbations, the frequency of asthma patients with SARS-CoV-2 infection is lower than that of asthma patients with rhinovirus infection (81). Chronic obstructive pulmonary disease (82) and patients with asthma prescribed high-dose ICS were associated with an increased risk of death (83). Also, patients with nonallergic asthma had worse clinical outcomes that patients with allergic asthma (84). Type 2 cytokines decreased the mRNA level of angiotensin converting enzyme 2 (ACE2), a receptor for SARS-CoV-2, in epithelial cells, which may reduce the risk of infection (85). In addition, ICS decreased the expression of ACE2 in alveolar epithelial cells (86). Furthermore, the use of ICS was associated with low expression of ACE2 (87). However, patients with asthma who were prescribed high-dose ICS were at an increased risk of death. This finding indicates that high-dose ICS is possibly associated with high risk of COVID-19-related death (83), although severity of asthma may influence the outcome of COVID-19 infection. A recent study demonstrated that biological therapeutics for severe asthma can be used safely, with low risk of developing severe COVID-19 (88, 89). Another report showed that patients with severe asthma treated with anti-IL-5 receptor antibody may be at low risk of developing severe COVID-19 (90, 91). Thus, therapeutics should be used to obtain better control of the disease symptoms in patients with severe asthma during the COVID-19 pandemic.

### 3.2 Role of eosinophils in COVID-19

Some clinical studies have indicated that blood eosinophil count (BEC) is a potential prognostic biomarker for COVID-19. In 140 patients hospitalized with COVID-19, more than half presented with eosinopenia during the early stage of infection (90, 92). A comparison of BEC between survivors and non-survivors during the disease course of COVID-19 showed that survivors had higher BEC than non-survivors during the recovery period (93). Among patients with asthma, eosinophils were not detected in the blood of 85% patients with COVID-19 on admission, and a BEC lower than 150/µl predicted a higher mortality rate during the disease course of COVID-19 (94). Autopsy of cases with severe COVID-19 demonstrated the absence of eosinophilic infiltration in the inflamed lung (95). Some studies have shown the activated status of blood eosinophils in COVID-19 patients. The number of blood eosinophils with high expression of CD62l increased on days 2‒6 of hospitalization. IFN-γ is suggested to be an activator of eosinophils during SARs-CoV-2 infection (96). Another report also demonstrated that CD62l-high eosinophils in severe COVID-19 cases expressed high levels of CXC chemokine receptor 4 (CXCR4) and programmed cell death 1-ligand 1 (PD-L1) (97). Interestingly, ECP and EDN concentrations in the sputum and BALF of patients with severe COVID-19 were higher in the first 10 days of severe infection than those in patients with a mild case (98). A case of suspected vasculitis, in which eosinophils accumulated around blood vessels in lung tissue with increased BEC, has been reported in COVID-19 (99). These findings suggest that eosinophils may play a protective role in COVID-19. Further studies are needed to confirm the role of eosinophils in the pathogenesis and progression of COVID-19.

## 4 Antiviral functions of eosinophils in viral infection

IFNs are produced as antiviral molecules during viral infection in the body. Among the IFNs, IFN-γ, a type 2 interferon, is a potent activator of eosinophils. Viruses are recognized by toll-like receptors (TLRs) 3/7/8/9 and specific types of pattern recognition receptors (PRRs) expressed on immune cells that recognize microorganisms. Retinoic acid inducible gene-I (RIG-I) and melanoma differentiation-associated protein 5 are RIG-I-like receptors (RLRs) that are also virus-associated molecules. Among these receptors of human eosinophils, TLR7 and RIG-I play major roles in the cellular response to viruses (100–102). Previous studies have shown that human and murine eosinophils are capable of capturing and inactivating viruses (17). Activated eosinophils release ECP and EDN, which possess ribonuclease activity. The ribonuclease activities of these granule proteins contribute to their antiviral effects on RNA viruses. Furthermore, eosinophils can synthesize fatty acid metabolites with antiviral activity. However, the significance of eosinophils *in vivo* as antiviral effector cells has not been completely elucidated. In the following sections, we discuss the eosinophil-related factors associated with antiviral activity.

### 4.1 IFN-γ

IFNs are proteins with virus-interfering effects that are categorized into type 1 (IFN-α and IFN-β), type 2 (IFN-γ), and type 3 IFNs (IFN-λ); type 1 IFNs show the strongest antiviral activity. In contrast, IFN-γ robustly enhances inflammatory responses and is mainly produced by Th1 cells, CD8+ T cells, macrophages, and natural killer (NK) cells. IFN-γ receptors, IFN-γR1 and IFN-γR2, mediate downstream signaling via the Janus kinase-signal transducer and activator of transcription 1 (JAK-STAT1) pathway (103). IFN-γ induces interferon-stimulated gene expression. Protein kinase R and adenosine deaminases acting on RNA (ADARs) function as antiviral proteins (104). Guanylate-binding proteins (GBPs) and tripartite motifs (TRIMs) with antiviral properties against RNA viruses, influenza virus and RSV, are also induced by IFNs (105–109). Eosinophils express functional IFN receptors, especially for IFN-γ. IFN-γ prolongs eosinophil survival more strongly than type 1 IFNs do (110–112). Blood eosinophils express CD69, an activation marker, via JAK2 upon IFN-γ stimulation (110). IFN-γ stimulation activates eosinophils that function as effector cells (113). IFN-γ stimulation induces ROS production and degranulation of eosinophils via mitogen-activated protein kinase (MAPK) (114, 115). Notably, eosinophil-derived IFN-γ enhances AHR in a murine model of asthma and stimulates the release of ECP from eosinophils (116). IFN-γ-induced antiviral activity of eosinophils has not yet been completely elucidated. IFN-γ-stimulated eosinophils exert antiviral activity via intracellular nitric oxide (NO) production (18). This promotes virus elimination in mouse models of parainfluenza infection. In addition, eosinophils bind to RV via intercellular adhesion molecule-1 (ICAM-1) and present viral antigens to RV-specific T cells to induce their IFN-γ production (117). Adhesion of eosinophils to ICAM-1 can further activate the functions of eosinophils including ROS production (118). Our research groups identified that eosinophils stimulated with IFN-α and IFN-γ upregulate the antiviral molecules GBPs and TRIMs. IFN-γ-stimulated eosinophils specifically upregulate expression of ICAM-1 and Fc gamma receptor-1A (FCGR1A) which may result in enhanced ICAM-1-mediated adhesion to rhinoviruses and enhanced inflammatory functions in response to virus with IgG cross linking (119). C-X-C motif chemokine ligand 10 (CXCL10), a ligand for CXCR3, is associated with IFN-γ. CXCL10 production induced by IFN-γ has been identified as a biomarker for rhinovirus-induced asthma exacerbations. CXCL10 stimulation increases ICAM-1 expression and reactive oxygen species (ROS) production in eosinophils, underscoring the pro-inflammatory role of IFN-γ in eosinophils (120, 121). However, further studies are required to elucidate the detailed mechanism.

### 4.2 TLR7

TLRs are a type of PRR that recognize pathogen-associated molecular patterns. TLRs are type I transmembrane proteins with external, transmembrane, and intracytoplasmic regions. TLRs of the intracytoplasmic region activates downstream signaling pathways, including nuclear factor-kappa B (NF-*κ*B), MAPK, and interferon regulatory factors (IRF)-3, to induce the expression of cytokines and chemokines, including IFNs (122). TLR3, TLR7, and TLR8 are localized intracellularly and recognize RNA viruses. TLR3 recognizes dsRNA, while TLR7 and TLR8 recognize single-stranded RNA (ssRNA), respectively (123). Among TLRs, eosinophils highly express TLR7. Stimulation with TLR7 ligand changes the adhesion molecule expression, ROS generation, cytokine production, and prosurvival pathways (100, 124). TLR7 expression in eosinophils was upregulated upon stimulation with IFN-γ but not with IL-4 and IL-5 (100). TLR7 signaling is mediated by p38-MAPK, phosphoinositide 3-kinase, extracellular signal-regulated kinase, and NF-*κ*B (124). In mouse models infected with RSV and parainfluenza virus, activated eosinophils thorough TLR7 eliminate these viruses via IRF-7-mediated induction of intracellular NO and eosinophil-associated ribonucleases (EAR)-1 and EAR-2, which possess ribonuclease activity (18, 19). Interestingly, TLR7 expression in immune cells is reduced in patients with asthma (125). In murine asthma models, administration of the TLR7 ligand suppressed allergic airway inflammation (126, 127). TLR7 governs IFN-related responses to rhinovirus and its expression is suppressed by IL-5-induced lung eosinophilia (128). These results suggested that eosinophil-expressing TLR7 exerts antiviral and/or anti-allergic effects that might be impaired in asthma.

### 4.3 RIG-I

RIG-I is a cytoplasmic RNA helicase that is a retinoic acid-induced RLR. RIG-I recognizes double-stranded viral RNA. RIG-I signals are transmitted by NF-*κ*B and IRF-3 to produce type 1 IFNs (129). RIG-I is expressed in virus-infected cells, including airway epithelial cells and macrophages. Human airway epithelial cells infected with rhinovirus express RIG-I via TLR3 (130). Eosinophils also express RIG-I and exert an RIG-I-dependent antiviral effect. In human eosinophils, α2–6 and α2–3-linked sialic acids reduce titers of the H1N1 influenza type A virus and express RIG-I mRNA while these precise mechanisms are not fully uncovered (20). In a murine model of fungus-sensitized asthma, exposure to a novel influenza virus (A/California/04/2009) increases intracellular RIG-I expression in bone marrow-derived eosinophils, leading to CD8+ T cell expansion (102). However, the mechanism underlying the RIG-I-mediated antiviral effect in eosinophils is unclear.

### 4.4 EDN and ECP

Eosinophils harbor abundant intracellular granule proteins, including MBP, EDN, ECP, and EPO (131). EDN and ECP exert antiviral effects on RNA viruses due to their ribonuclease activity (16). Sequences orthologous to human EDN and ECP have been identified in higher primate genomes. EDN/RNase2 and its divergent ortholog, mouse eosinophil-associated RNases (mEars), are prominent secretory proteins of eosinophils within the RNase A-type ribonuclease family (132). A previous study has revealed that the antiviral activity of EDN was higher than that of ECP (133). As mentioned above, murine eosinophils recognize the RSV via the TLR7-Myd88 system, express the genes encoding granule proteins (EAR1 and EAR2), and release ECP (19). Also, recombinant human eosinophil-derived neurotoxin/RNase 2 functions as an effective antiviral agent against RSV (134) However, the importance of granule proteins in RNA virus clearance in human remains unclear.

### 4.5 Specialized pro-resolving mediator (SPM)

**Figure 1:**
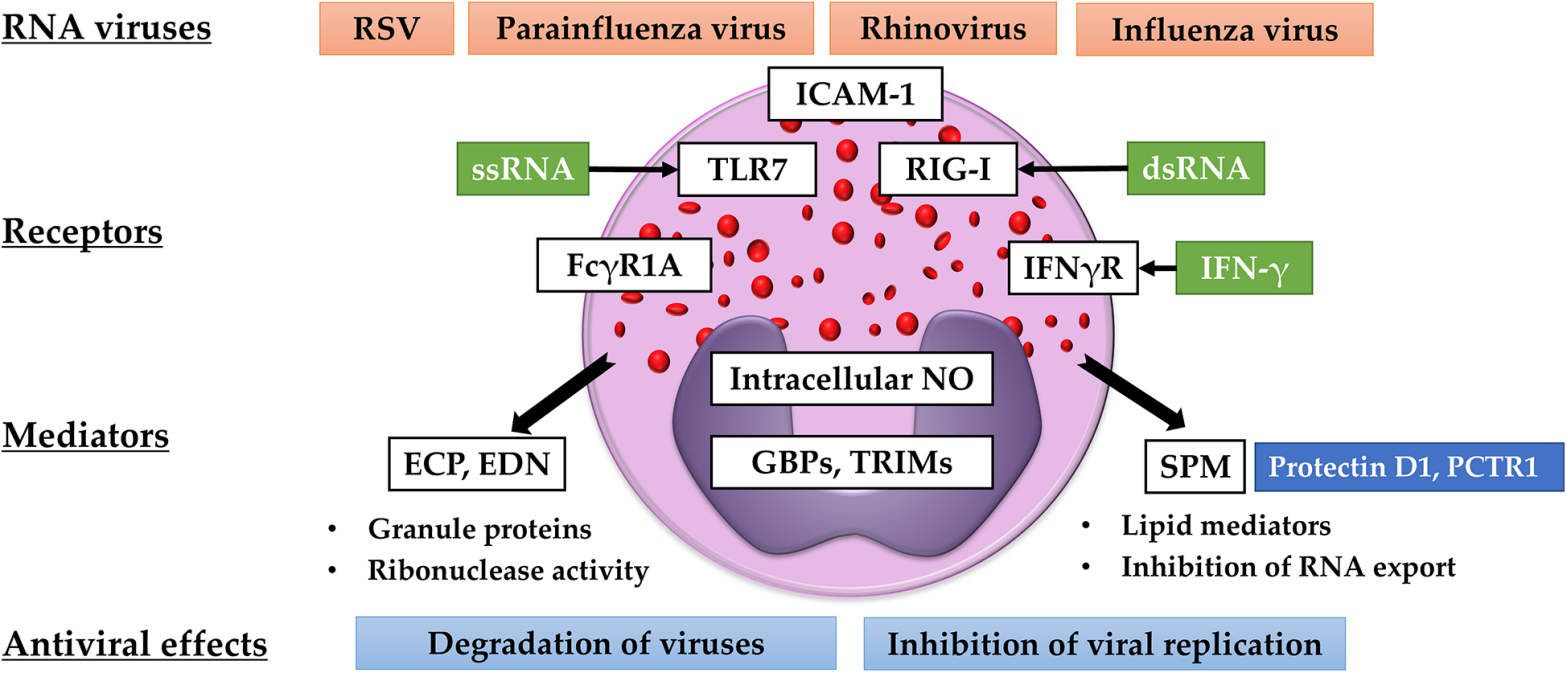
Antiviral activities of eosinophils. Eosinophils recognize RNA viruses via TLR-7 and RIG-I. The expression of ICAM-1, FCGR1A, and TLR-7 is upregulated by IFN-γ. Eosinophils produce ribonucleases (ECP and EDN), intracellular NO, SPMs, GBPs, and TRIMs. These mediators are believed to degrade viruses and inhibit viral replication and transport.

Eosinophils can synthesize fatty acid-derived bioactive mediators (135). During allergic inflammation, eosinophils produce large amounts of cys-LTs that are converted from arachidonic acid released from nuclear membranes. Our research revealed that human eosinophils stimulated with IL-5 produce LTD_4_, a ligand with high affinity to CysLT1 and CysLT2, and the combined stimulation with IL-5 and IL-4 further augments its production (136). Cys-LTs induce airway constriction, increase vascular permeability, and enhance mucus production and accumulation of inflammatory cells (137). On the other hands, eosinophils can synthesize SPMs via the fatty acid metabolizing enzyme, 15-lipoxygenase (15-LOX), which inhibits allergic airway inflammation and promotes the resolution of inflammation (138, 139). In murine tissue, tissue-resident eosinophils specifically express this enzyme, which possibly contributes to the maintenance of homeostasis (140, 141). Previous studies have shown that SPMs also play important roles in infection (142). The production of a docosahexaenoic acid (DHA)-derived SPM, protectin D1 (PD1), decreased following lethal influenza virus infection (143, 144). In a murine model of influenza virus infection, PD1 prevented fatal infection by inhibiting viral replication in airway epithelial cells (144). Results of an *in vitro* lipid screening assay demonstrated that 15-LOX-derived metabolites, including PD1, exerted similar inhibitory effects on viral replication (144). A recent study also showed that PD1 and another SPM, protectin conjugates in tissue regeneration 1 (PCTR1), induced the production of IFN-λ from airway epithelial cells and inhibited RSV replication (145). These results indicated that SPMs may function as antiviral molecules *in vivo*. Our study showed that human eosinophils are capable of synthesizing sufficient amounts of PD1 (37, 146). Interestingly, blood eosinophils isolated from patients with severe asthma have reduced capacity of producing PD1 and other 15-LOX-derived metabolites (37). Reduced PD-1 production may result from impaired DHA utilization in eosinophils and defective 15-LOX metabolic synthesis, although no change in 15-LOX was observed in eosinophils stimulated with IL-4 or IL-4 plus IL-5 compared to non-stimulated cells (136). These findings suggest that antiviral activity via 15-LOX metabolism in eosinophils may be reduced in refractory eosinophilic diseases, and further investigation is required to elucidate the balance between the pro-inflammatory and anti-inflammatory roles of eosinophils. The antiviral activities of eosinophils have been summarized in Figure 1.

## 5 Conclusion

Eosinophils accumulate in the airways of patients with asthma due to type 2 cytokine-dependent inflammation and enhance type 2 inflammation with tissue injury. Eosinophils can also be activated by IFN-γ and can recognize RNA viruses via TLR7 and RIG-I (18, 20). They can clear viruses via ECP and EDN granule proteins and intracellular NO (16, 18). Interestingly, non-eosinophilic exacerbations triggered by viral or bacterial infections were observed in patients treated with mepolizumab (147), an anti-IL-5 antibody, suggesting that removal of eosinophils may cause expansion of virus and/or bacteria. However, patients with asthma have reduced capacity of producing IFNs and are vulnerable to viral infections (52, 54). In addition, the antiviral activity of eosinophils is impaired in asthma patients, especially in patients with severe asthma (17). Our study also reported that the expression of antiviral molecules up-regulated by IFN-γ in human eosinophils was attenuated by co-stimulation with IFN-γ and IL-5 (119). Further investigations are required to better understand the role of eosinophils in viral infection. Additionally, novel therapeutic strategies for severe asthma are required to suppress allergic inflammation and enhance antiviral defense.
